# Responses of soil fertility indicators and fungi community diversity to fertilization strategies in legume-grass mixtures

**DOI:** 10.3389/fpls.2025.1579011

**Published:** 2025-05-08

**Authors:** Jingru Chen, Xiaoshan Chen, Li li Nan

**Affiliations:** ^1^ Key Laboratory of Grassland Ecosystem of Ministry of Education, College of Pratacultural Science, Gansu Agricultural University, Lanzhou, China; ^2^ Key Laboratory of Forage Germplasm Innovation and New Variety Breeding of Ministry of Agriculture and Rural Affairs (Co-sponsored by the Ministry and Gansu Province), Lanzhou, China

**Keywords:** fertilization, legume-grass mixtures, fungi community, soil fertility indicators, rhizosphere soil

## Abstract

**Introduction:**

Alfalfa-grass binary mixtures outperformed monocultures in nutrient use, soil nutrient content, and biomass yield. Nonetheless, the impact of fertilization strategies on fungal community composition and ecological functions in legume-Grass mixtures remains under-researched. This study aimed to explore the effects of different fertilization strategies on soil fungal community distribution and soil environmental characteristics.

**Methods:**

A field experiment in Gansu, China, has been conducted to explore the effects of five different fertilization strategies—no fertilization (CK), three partial fertilization methods (+PK, +NK, +NP), and balanced fertilization (+NPK)—on fungal population richness, community composition, and soil environmental drivers. Rhizosphere soils from the five treatments were sampled and investigated using high-throughput ITS sequencing.

**Results:**

Compared to CK, +NPK led to higher soil capabilities (*P*< 0.05), soil organic matter (SOM), available nitrogen (AN), available phosphorus (AP), and available potassium (AK) increased by an average of 29.7 %, 42.3 %, 101.2 %, and 24.3 %, respectively; alkaline phosphatase (APA), catalase (CAT), and sucrase (SA) increased by an average of 56.6 %, 31.8 %, and 46.7 %, respectively; soil microbial biomass carbon (SMBC), soil microbial biomass nitrogen (SMBN), and soil microbial biomass phosphorus (SMBP) increased by an average of 64.8 %, 65.1 %, and 60.4 %, respectively. The dominant fungi in the rhizosphere soil were Mortierellomycota and Ascomycota, accounting for 82.2%-92.3%. The fungal species richness was the highest in the +PK treatment. From the NMDS and RDA analysis, it can be discerned that SA, AK, and CAT were the key environmental factors influencing the structure of the inter-root soil fungal community in alfalfa; CAT and SOM were the key environmental factors influencing the structure of the inter-root soil fungal community in awnless brome.

**Discussion:**

Our findings investigated the optimal fertilizer strategy for legume-Grass mixtures. Results provided a technical basis for scientific fertilizer application and development of local mixed grassland ecosystems.

## Introduction

The Hexi Corridor in Gansu Province serves as a crucial agricultural and pastoral production base in China. However, the use of unsuitable grassland management techniques and grazing methods results in considerable deterioration of the natural grasslands in this area (Jia et al., 2022). The establishment of artificial grasslands is emerged as a pivotal strategy for promoting intensive livestock production within grassland ecosystems (Chen et al., 2021). The region is characterized by nutrient-poor soils and a fragile ecological environment. Furthermore, mowing practices exacerbate soil nutrient depletion through the removal of substantial quantities of mineral elements (Chi et al., 2023). In the establishment of artificial grasslands, relying solely on soil nutrient provision is insufficient to maximize the production potential of high-quality forage grasses. Recent studies have demonstrated that legume-grass mixed pastures exhibit superior productivity, enhanced forage quality, improved soil fertility, and optimized resource utilization efficiency compared to monoculture systems (Wang et al., 2024a). Within this cultivation system, the legume component plays a crucial role in augmenting the carbon sink in pasture soil, enhancing protein self-sufficiency, and reducing production costs by eliminating the need for nitrogen fertilizer in forage grass production (Lüscher et al., 2014). Concurrently, the grass component contributes to increased total biomass yield and helps mitigate issues such as weed encroachment, legume lodging, and bloating (Tahir et al., 2022).

Additionally, the presence of species diversity generates complementarity, insurance, and sampling effects, which are key factors in enhancing the biomass output and stability of mixtures (de Souza et al., 2024; Liu et al., 2024a). Niche complementarity, a beneficial interaction between different species, allows each species to utilize resources differently or access distinct forms of resources (Jensen et al., 2025). For instance, alfalfa-grass binary mixtures possess distinct root systems (i.e., tap root *vs*. fibrous), enabling both species to access soil nutrients at varying depths. Alfalfa (*Medicago sativa*), a premium forage legume, when paired with perennial grasses, enhances cattle protein intake and improves pasture soil health (Bhandari et al., 2020). Awnless brome (*Bromus inermis*) is known for its nutritional value, palatability, cold resistance, and durability (Li et al., 2022b). Both are the primary cultivated forage grasses in northern China (Liu and Gong, 2022), which were the most common binary mixtures in Gansu province. In light of the ongoing degradation of natural grasslands, mixed cultivated grassland can support the stable and sustainable production of animal husbandry. Studies show that the rational application of nitrogen, phosphorus, and potassium fertilizers has an important impact on the growth and development of legume-grass mixed grassland (Tahir et al., 2023).

Fungi are integral to nutrient cycling in soil ecosystems (Jia et al., 2020). As the primary decomposers in the soil food web, fungi can break down complex organic matter, releasing essential nutrients such as carbon (C) and nitrogen (N) for plant uptake (Ren et al., 2024). Recent studies have demonstrated that fungi can degrade organic substances that are resistant to decomposition by other microorganisms (Ma et al., 2024). Additionally, they influence the physical and chemical properties of soil, contribute to nutrient cycling, enhance soil fertility, and play a vital role in maintaining soil quality and promoting plant growth (Bai et al., 2019). Previous research on the impact of fertilization methods on microbial communities demonstrates that different fertilization strategies can alter the structure and activity of soil microbial populations (Mishra et al., 2024). In the context of mixed planting, these changes are often attributed to the influence of root exudates from different plant species on soil chemical properties, which in turn affect the composition of the fungal community (Fan et al., 2024). However, despite the significant progress in understanding soil microbial ecology, there remain gaps in knowledge regarding the effects of various fertilization strategies on soil nutrients and microbiological characteristics in legume-Grass mixtures (Yeboah et al., 2021).

The present study on forage production in this area mainly involves grazing, nitrogen addition, and other variables (Li et al., 2022a). The influence of fertilizer on soil biological parameters and fungal community composition in legume-Grass mixtures in the Hexi Corridor has yet to be fully investigated. In this study, we conducted a comprehensive investigation in a desert irrigation area of the Hexi Corridor, where legume-Grass mixtures were planted. Our research aims to: (i) precisely quantify the responses of soil nutrient content, enzyme activity, and biological characteristics to different fertilization treatments; (ii) comprehensively evaluate the response trends of fungal community structure and abundance to fertilization treatments in legume-Grass mixtures; and (iii) to clarify the effects of fertilization on the composition of fungi in legume-Grass mixtures in the Hexi Corridor. By achieving these objectives, we seek to provide a scientific basis for optimizing fertilization strategies in legume-Grass mixtures, thereby promoting sustainable development in local grassland ecosystems.

## Materials and methods

### Experimental sites and design

**Figure 1:**
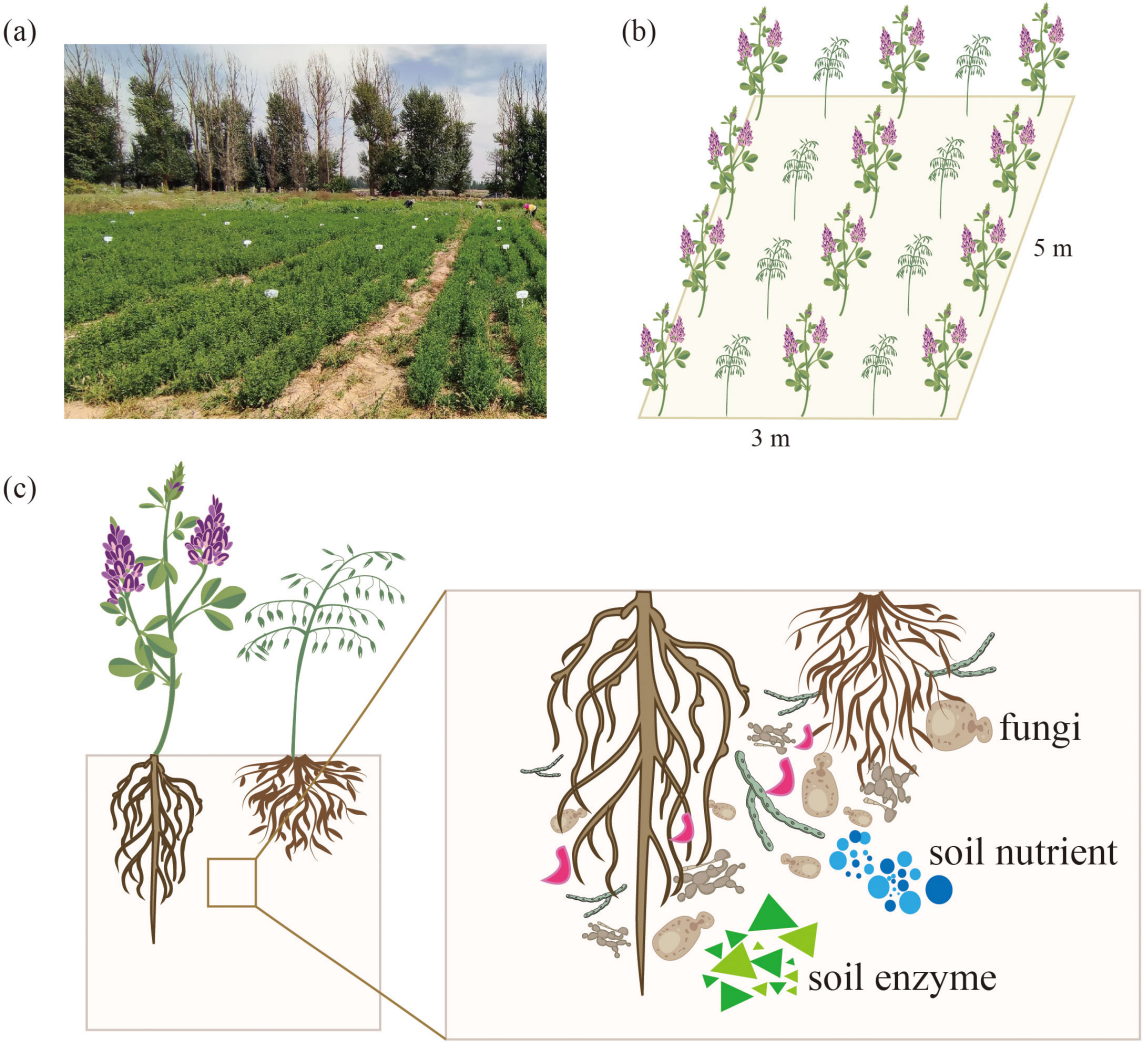
Schematic diagram of field planting. **(a)** Field photo, **(b)** Schematic of field planting, **(c)** Schematic of rhizosphere soil.

This study was conducted at the Wuwei Pasture Experiment Station in Gansu Province, China. The region experiences a temperate arid desert climate and sandy loam soil. More details regarding the sites are outlined in **Table 1** below. The experiments were conducted in a completely randomized design with five treatments and three replicates, constituting fifteen plots. Each plot covered an area of 15 m^2^ (3 m × 5 m), with the two plots separated by 1 m and bordered by ridges (**Figure 1**). The optimal local fertilization rate was calculated according to the fertilization formula and referred to the fertilization scheme for alfalfa forage production in this area by Wang Xinyou (Wang et al., 2021) and Wu Yong (Wu et al., 2020). The optimum fertilization in this area was determined as follows: N 120 kg/ha, P 210 kg/ha, K 90 kg/ha. Therefore, the five different fertilization strategies were: CK, +PK, +NK, +NP, +NPK. The test fertilizers were CO(NH_2_)_2_ (N 46%), Ca(H_2_PO_4_)_2_ (P 12%) and K_2_O (K 51%). The alfalfa (*M. sativa* ‘Gannong’ No. 3, or GN3) and the grass forage *Bromus inermis* ‘Yuanye’ were adopted as planting material (‘G’ denotes alfalfa, while ‘Y’ represents awnless brome). On May 2, 2022, alfalfa and awnless brome were sown in a 1:1 row ratio at sowing rates of 10 kg/ha and 15 kg/ha, respectively. The row spacing for the mixed planting of alfalfa and awnless brome was 0.3 m. Before cultivating the legume-grass mixture, phosphorus and potassium fertilizers were evenly applied. Nitrogen fertilizer was applied on three occasions: once at the time of sowing and after each cutting, followed by thorough irrigation. Drip irrigation was used as the irrigation method. Winter irrigation occurred on October 20, while spring irrigation occurred on May 1, each year. Additional irrigation was timed after each cutting (August 14, 2022; June 9, 2023; July 17, 2023; September 4, 2023), with each irrigation amounting to approximately 1120 m^3^/hm^2^. The experiment was conducted during the second year of cultivation and the rhizosphere soil indices for the second crop were determined.

**Table 1:** Climatic conditions, locations, and soil properties of the sites.

Parameters	Wuwei Pasture Experiment Station
pH	8.7
Total nitrogen (g/kg)	1.07
Total phosphorus (g/kg)	3.32
Soil organic matter (g/kg)	8.65
Available nitrogen (mg/kg)	81.8
Available phosphorus (mg/kg)	13.24
Available potassium (mg/kg)	93.95
Temperature	7.2°C
Rainfall	150 mm
GPS coordinates	
Latitude	37°55'N
Longitude	102°40'E

### Sample sampling

On July 28, 2023, in each experimental plot, five leguminous plants and five grass plants were randomly selected in each experimental plot. The entire plant was excavated and the soil surrounding the roots system was carefully removed. The soil tightly adhered to the root system (rhizosphere soil) was brushed off, and then the soil samples of 5 plants of the same family in each fertilization treatment were combined to form a rhizosphere soil sample of fertilization treatment. Such soil samples were repeated for 3 times (5 treatments, 2 species, 3 replicates, 5×2×3 = 30). The samples were transported to the laboratory through a sampling box at -4°C after being cleaned by removing plant roots, stones, and other debris. The soil samples were then divided into three parts: one part was sieved through a 2 mm mesh and stored at -80°C for total soil microbial DNA extraction; the other part was stored at 4°C for the determination of soil microbial biomass; and the remaining part was air-dried indoors, sieved through 1 mm, and used for the determination of soil enzyme activity.

### Soil sample analyses

#### Soil nutrient content and microbial biomass

Soil organic matter (SOM) was measured by the potassium dichromate volumetric method (external heating) (Xu et al., 2024). Available potassium (AK), available phosphorus (AP), and available nitrogen (AN) were determined by alkaline dissolution diffusion method, molybdenum antimony colorimetry, and Ammonium acetate (NH_4_OAc) extraction-flame spectrophotometry (Wang et al., 2023). Simply put, weigh 2 g of air-dried soil samples through a 1 mm sieve and put them into the outer cavity of the diffusion dish. After the soil sample was evenly paved, a 2 mL H_3_BO_3_ indicator was injected into the outer cavity of the diffusion dish. The NH_3_ in the intracavity absorption solution was removed and titrated with 0.01 mol/L 0. 5 H_2_SO_4_ standard solution. For AK determination, 2.5 g of air-dried soil samples passed through a 1mm sieve were weighed, 50 mL of cold 2 mol/L HNO_3_ was added, oscillated for 30 min, then filtered with quantitative filter paper immediately, and determined with potassium standard series solution on flame photometer. Air-dried soil (soil: water, 1:5) was used to determine pH (Huang et al., 2021).

Soil microbial biomass carbon (SMBC), soil microbial biomass nitrogen (SMBN), and soil microbial biomass phosphorus (SMBP) were assessed using chloroform fumigation. Both fumigated and unfumigated soil samples were extracted with a 0.5 mol/L K_2_SO_4_ solution. The carbon content of the microbial biomass in the extract was determined through titration with FeSO_4_ solution, with a conversion coefficient (KC) set at 0.38 (Fan et al., 2024). Microbial biomass nitrogen and phosphorus were adjusted using extraction coefficients of 0.45 and 0.40, respectively (Ma et al., 2023; Sikorski et al., 2022). Based on the soil water content measured using the weighing method, a correction factor for soil samples with varying moisture levels is determined to standardize them to a common humidity condition. The adjusted soil microbial biomass is then calculated by multiplying the actual measured microbial biomass data by this correction factor.

#### Soil enzyme activity

Alkaline phosphatase (APA) activity was assessed using disodium phenyl phosphate (Cui et al., 2024). To begin, weigh 5 g of a fresh soil sample and add 2.5 mL of toluene. Shake the mixture for 15 minutes, then incorporate 20 mL of a solution containing 0.5 parts by weight of disodium phenyl phosphate. The mixture was allowed to ferment at 37°C for two hours (constant temperature). Following the fermentation period, 100 mL of 0.3% alum solution was added, and then the mixture and the specific volume of the filtrate were extracted. The phenol content of the filtrate was determined using colorimetry based on a standard curve. After 24 hours, the phenol content per gram of soil was measured, serving as an indicator of phosphatase activity.

Catalase (CAT) activity was assessed using KMnO_4_ titration (Gao et al., 2023). In brief, dry soil was weighed and mixed with 40 mL of distilled water and 5 mL of 0.3% H_2_O_2_. The mixture was sealed with a membrane and oscillated at 120 times/min for 20 minutes. Subsequently, 10 mL of 3 mol/L H_2_SO_4_ was immediately added to halt the reaction and the mixture was filtered. A volume of 25 mL of the filtrate was titrated with 0.1 mol/L KMnO_4_ until a reddish endpoint was reached. To quantify catalase activity, the amount of 0.1 mol/L KMnO_4_ consumed per gram of soil per hour was calculated. Sucrase (SA) activity was determined using 3,5-dinitro salicylic acid colorimetry (Huang et al., 2021). Specifically, 5 g of air-dried soil was weighed, and 15 mL of an 8% sucrose solution, 5 mL of phosphate buffer (pH 5.5), and 5 drops of toluene were added. The mixture was thoroughly shaken and incubated at 37°C for 24 h (constant temperature). After incubation, 1 mL of the filtrate was collected and 3 mL of 3,5-dinitrosalicylic acid was added to a volumetric flask. This mixture was heated in boiling water for 5 minutes, cooled, diluted, and then analyzed colorimetrically at 508 nm. After 24 h, invertase activity was measured in milligrams of glucose per gram of soil.

### The extraction and sequencing of entire soil DNA

A total of 30 samples (5 treatments×2 species×3 replicates) were subjected to sequencing according to the following protocol. DNA was isolated using an isolation kit (MO BIO Laboratories Inc., Carlsbad, CA, USA) from 0.25 g of soil. To estimate the abundance of fungi, copy numbers of fungal ITS1 genes were quantified. Subsequently, the PCR products were purified, and the SMRTbell library was evaluated before sequencing the libraries using the PacBio Sequel platform. Biomarker Technologies Corporation performed all the procedures. The raw sequences were deposited in the NCBI SRA database with the accession number PRJNA1193339.

### Bioinformatics analysis and data preprocessing

Before generating cyclic consensus sequencing (CCS) data, the original sub-reading code was corrected using SMRT Link version 8.0. Chimeric sequences in these readings were removed with UCHIME version 8.1 to obtain high-quality CCS readings. The readings were then mass-filtered to produce refined CCS data (Edgar et al., 2011). The UPARSE-OUT algorithm was employed to generate operational taxonomic unit (OTU) clusters with a 97% feature threshold (Edgar, 2013). Using the SILVA classification database, OTUs were classified based on high-quality CCS readings (Quast et al., 2012). Subsequently, various taxonomic levels were analyzed to study the taxonomic distribution and community structure. Analysis of variance (ANOVA) was conducted to examine significant differences in alpha diversity among the samples. Non-metric multidimensional scaling (NMDS) was utilized as a suitable ranking method for ecological research, as it better reflects the nonlinear structure of ecological data.

### Statistical analysis

Soil nutrient content, enzyme activity, and biological characteristics were analyzed by one-way ANOVA, and these data were expressed by the mean ± standard deviation (SD) of three repeated samples. SPSS 26.0 was used for all statistical analysis. Generate bar graphs using GraphPad Prism version 9.0. The alpha diversity of microorganisms, including Ace, Chao 1, and Shannon, was calculated using the MOTHUR version 1.30. 1 software. The NMDS analysis was performed using BMKCloud (www.bioloud.net). The relationship between environmental factors and bacterial community structure was tested by redundancy analysis (RDA) using CANOCO 5.0 for Windows (Monte Carlo replacement test, *P*=0.001; Microcomputer Power, United States).

## Results

### Effect of fertilization strategies on soil properties, enzyme activities, and microbial biomass

#### Soil properties

**Figure 2:**
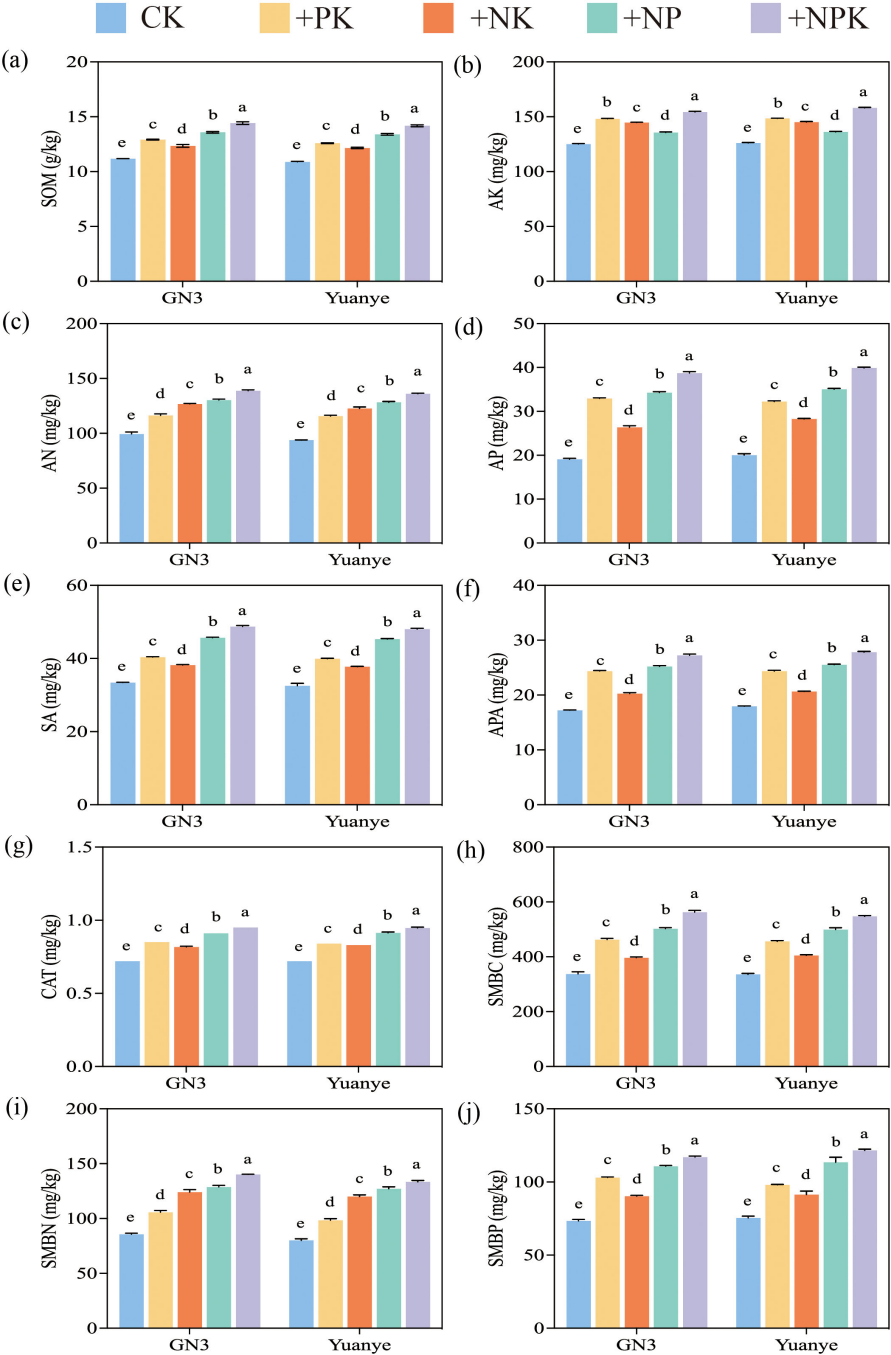
Soil properties, enzyme activities, and microbial biomass of Legume-Grass mixtures under fertilization treatment. **(a–j)** represent SOM (soil organic matter), AK (available potassium), AN (available nitrogen), AP (available phosphorus), SA (sucrase), APA (alkaline phosphatase), CAT (catalase), SMBC (soil microbial biomass carbon), SMBN (soil microbial biomass nitrogen), and SMBP (soil microbial biomass phosphorus) of rhizosphere soil respectively. In the treatment name, “G” represents *M. sativa* ‘Gannong’ No.3, while “Y” represents *Bromus inermis* ‘Yuanye’. CK (N 0 kg/ha, P 0 kg/ha, K 0 kg/ha), +PK (N 0 kg/ha, P 210 kg/ha, K 90 kg/ha), +N2K2 (N 120 kg/ha, P 0 kg/ha, K 90 kg/ha), +N2P2 (N 120 kg/ha, P 210 kg/ha, K 0 kg/ha), and +NPK (N 120 kg/ha, P 210 kg/ha, K 90 kg/ha). Different lowercase letters indicate significant differences between the treatments at a significance level of *P* < 0.05.

Under the four different fertilization treatments, the levels of SOM, AN, and AK in the rhizospheres of alfalfa and awnless brome were significantly higher than those in the control group (CK) (*P* < 0.05) (**Figure 2**). The results for the +NPK treatment were the most pronounced. Compared to the CK treatment, the SOM, AN, AP, and AK contents of the alfalfa rhizosphere soil under +NPK treatment were 29%, 40%, 103%, and 23% higher, respectively. Similarly, the analysis of nutrient content in the rhizosphere soil of the awnless brome indicated that SOM, AN, AP, and AK increased by 30.3%, 45.1%, 99.0%, and 25.4%, respectively, under the +NPK treatment. Overall, there were significant differences between the various treatments. Furthermore, the ranking of SOM and AP contents was as follows: +NPK > +NP > +PK > +NK. The ranking for AN content was: +NPK > +NP > +NK > +PK. In contrast, the order of AK content was +NPK > +PK > +NK > +NP.

#### Soil enzyme activities

In the rhizosphere soil of alfalfa, the activities of CAT, APA, and SA were measured at 0.72-0.95 mg/g, 17.22-27.24 mg/g, and 33.40-48.73 mg/g, respectively. In the rhizosphere soil of awnless brome, the activities of CAT, APA, and SA were recorded at 0.72-0.95 mg/g, 17.96-27.83 mg/g, and 32.55-48.04 mg/g, respectively (**Figure 2**). Furthermore, enzyme activities across the four fertilization treatments were significantly higher than those observed in the CK treatment (*P* < 0.05). Compared with the CK treatment, the activities of CAT, APA, and SA in the rhizosphere soil of alfalfa treated with +NPK increased by 58.2%, 31.8%, and 45.9%, respectively. Similarly, in the rhizosphere soil of awnless brome treated with +NPK, the activities of CAT, APA, and SA increased by 55.0%, 31.8%, and 47.6%, respectively. The ranking of enzyme activity was as follows: +NPK > +NP > +NK > +PK.

#### Soil microbial biomass

The contents of SMBC, SMBN, and SMBP in the rhizosphere soils of alfalfa and awnless brome were the highest under +NPK treatment (**Figure 2**). These levels were significantly higher than those observed under CK treatment, with average increases of 63% and 64%, respectively. In the rhizosphere of alfalfa treated with +NPK, the contents of SMBC, SMBN, and SMBP were 66.8%, 63.7%, and 59.2% higher than those in the CK treatment, respectively. Similarly, in the rhizosphere of awnless brome treated with +NPK, the contents of SMBC, SMBN, and SMBP were 55.9%, 62.9%, and 66.5% higher than those in the CK treatment, respectively. The results also indicated that the concentrations of SMBC and SMBP followed the order: +NPK > +NP > +PK > +NK, while the contents of SMBN followed the order: +NPK > +NP > +NK > +PK. Additionally, the contents of SMBC, SMBN, and SMBP were significantly different among the various fertilization treatments (*P* < 0.05).

### Analysis of fungal diversity in different fertilization treatments

Illumina high-throughput sequencing successfully identified 358195 fungal ITS1 regions. As shown in **Table 2**, sample coverage ranged from 98% to 99%. This showed that the index information can better reflect the diversity of fungal communities, better classify and study fungal communities, and then understand their distribution and change rules. In the alfalfa community, the number of fungal OUT in +NPKG treatment was the highest, which was slightly higher than in other fertilization treatments. In the awnless brome community, the number of fungal OUT was the highest in +NPY treatment. The results of the Alpha diversity index showed that the +PKG treatment had the highest fungal species richness, and the +NPG treatment had the highest fungal species diversity. In the awnless brome community, the fungal species richness of the +PKY treatment was the highest, and the fungal species diversity of the CKY treatment was the highest.

**Table 2:** Alpha diversity of the soil fungal community associated with legume and grass on the basis of Illumina high-throughput.

Treatment	Fungal communities					
	OTU	ACE	Chao1	Simpson	Shannon	Coverage(%)
CKG	424	854.42±42.43a	683.17±34.54a	0.84±0.03abc	4.02±0.42bc	98.2
+PKG	441	751.22±29.63b	690.64±24.53a	0.87±0.02abc	4.35±0.51ab	98.1
+NKG	438	544.24±41.88fg	501.13±5.99c	0.86±0.02abc	4.15±0.28bc	98.9
+NPG	460	657.63±44.33cd	615.84±24.14b	0.91±0.03a	4.93±0.18a	98.8
+NPKG	463	483.25±40.56g	461.77±40.77cd	0.86±0.02abc	4.25±0.17bc	99.0
CKY	491	609.26±37.87de	616.36±22.24b	0.90±0.01ab	5.04±0.17a	98.9
+PKY	463	675.63±44.33c	679.93±24.07a	0.84±0.03bc	4.64±0.43ab	98.3
+NKY	482	529.74±20.11fg	471.90±41.91cd	0.83±0.05c	4.07±0.48bc	98.9
+NPY	500	589.12±33.81ef	485.68±5.74cd	0.81±0.02cd	3.81±0.35c	98.8
+NPKY	489	496.49±32.09g	449.77±21.04d	0.76±0.07d	3.62±0.35c	99.0

Data represent mean ± SD, different lowercase letters in the same column indicate significant differences between the groups at a significance level of *P*< 0.05. In the treatment name, "G" represents *M. sativa* ‘Gannong' No.3, while "Y" represents *Bromus inermis* ‘Yuanye'. CK (N 0 kg/ha, P 0 kg/ha, K 0 kg/ha), PK (N 0 kg/ha, P 210 kg/ha, K 90 kg/ha), NK (N 120 kg/ha, P 0 kg/ha, K 90 kg/ha), NP (N 120 kg/ha, P 210 kg/ha, K 0 kg/ha), and NPK (N 120 kg/ha, P 210 kg/ha, K 90 kg/ha).

### Changes in soil fungal community abundance and composition

**Figure 3:**
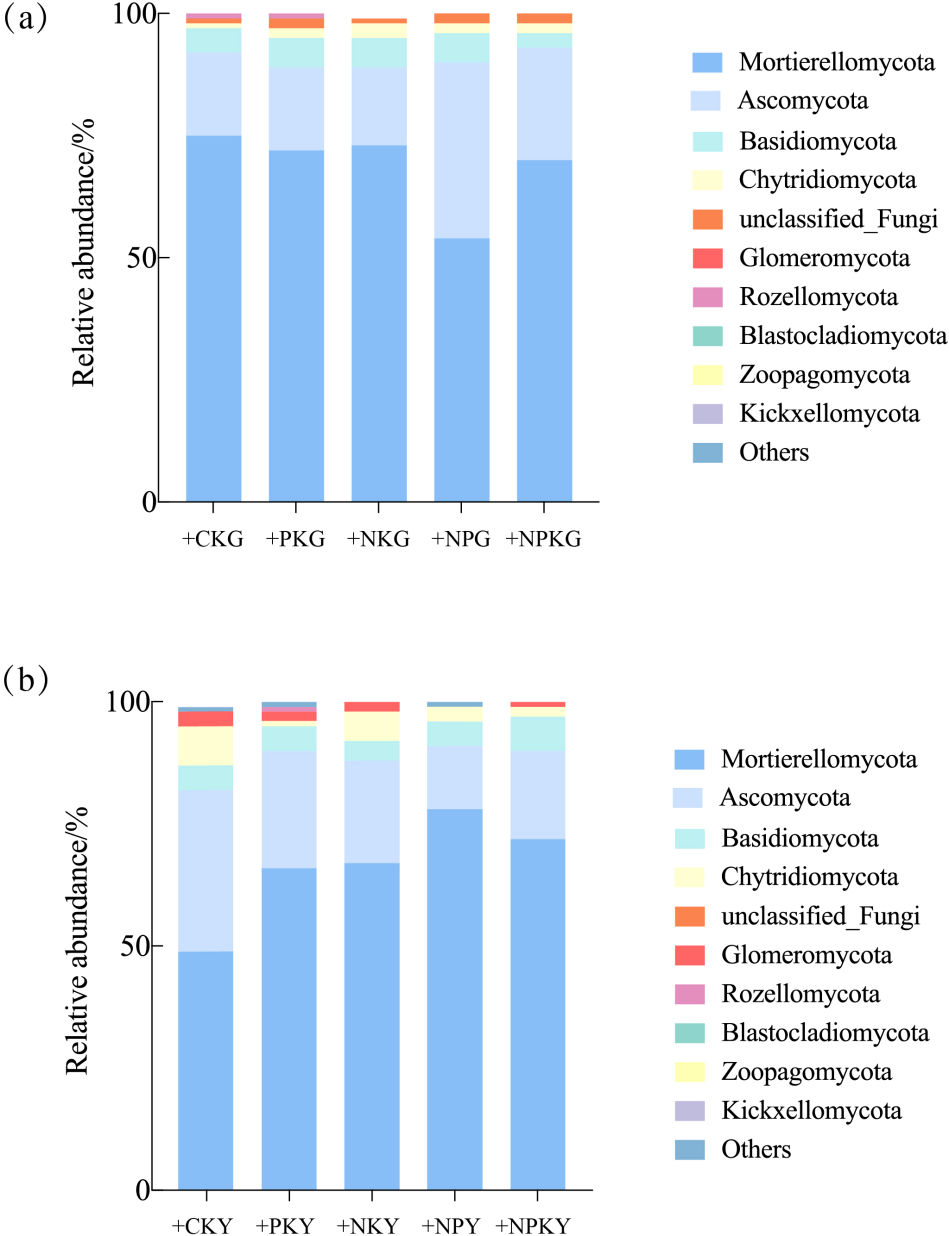
Relative abundance of fungal communities of Legume-Grass mixtures. **(a, b)** represent the phylum level of alfalfa and awnless brome, respectively. In the treatment name, “G” represents *M. sativa* ‘Gannong’ No.3, while “Y” represents *Bromus inermis* ‘Yuanye’. CK (N 0 kg/ha, P 0 kg/ha, K 0 kg/ha), +PK (N 0 kg/ha, P 210 kg/ha, K 90 kg/ha), +N2K2 (N 120 kg/ha, P 0 kg/ha, K 90 kg/ha), +N2P2 (N 120 kg/ha, P 210 kg/ha, K 0 kg/ha), and +NPK (N 120 kg/ha, P 210 kg/ha, K 90 kg/ha).

The dominant phyla were Mortierellomycota, Ascomycota, Basidiomycota, Chytridiomycota, Glomeromycota, Rozellomycota, Blastocladiomycota, Zoopagomycota, and Kickxellomycota, accounting for 95.5%-98.1% (**Figure 3**). The analysis revealed that the microbial community structure in the alfalfa and awnless brome rhizosphere soils was similar at the phylum level, with the fungi showing a consistent relative abundance of > 1% and no change in order.

For the alfalfa fungal community, the dominant phylum were Mortierellomycota, Ascomycota, Basidiomycota, and Chytridiomycota, with relative abundance ranging from 96.9% to 98.1%. The relative abundance of Mortierellomycota decreased by 7.49% after the +NPK treatment, as well as a 35.65% decrease in Basidiomycota, compared to the CK treatment. Inversely, Ascomycota and Chytridiomycota experienced an increase in relative abundance, with a rise of 33.64% and 121.62%. For the awnless brome community, the dominant phylum was the same as the alfalfa community, with relative abundance ranging from 95.5% to 98.1%. Compared to the CK treatment, the +NPK treatment resulted in a higher relative abundance of Mortierellomycota and Basidiomycota by 45.67% and 27.82%. Oppositely, the relative abundance of Ascomycota and Chytridiomycota decreased by 46.54% and 79.97%.

### NMDS analysis and RDA analysis of soil fungal community structure under different fertilization treatments

**Figure 4:**
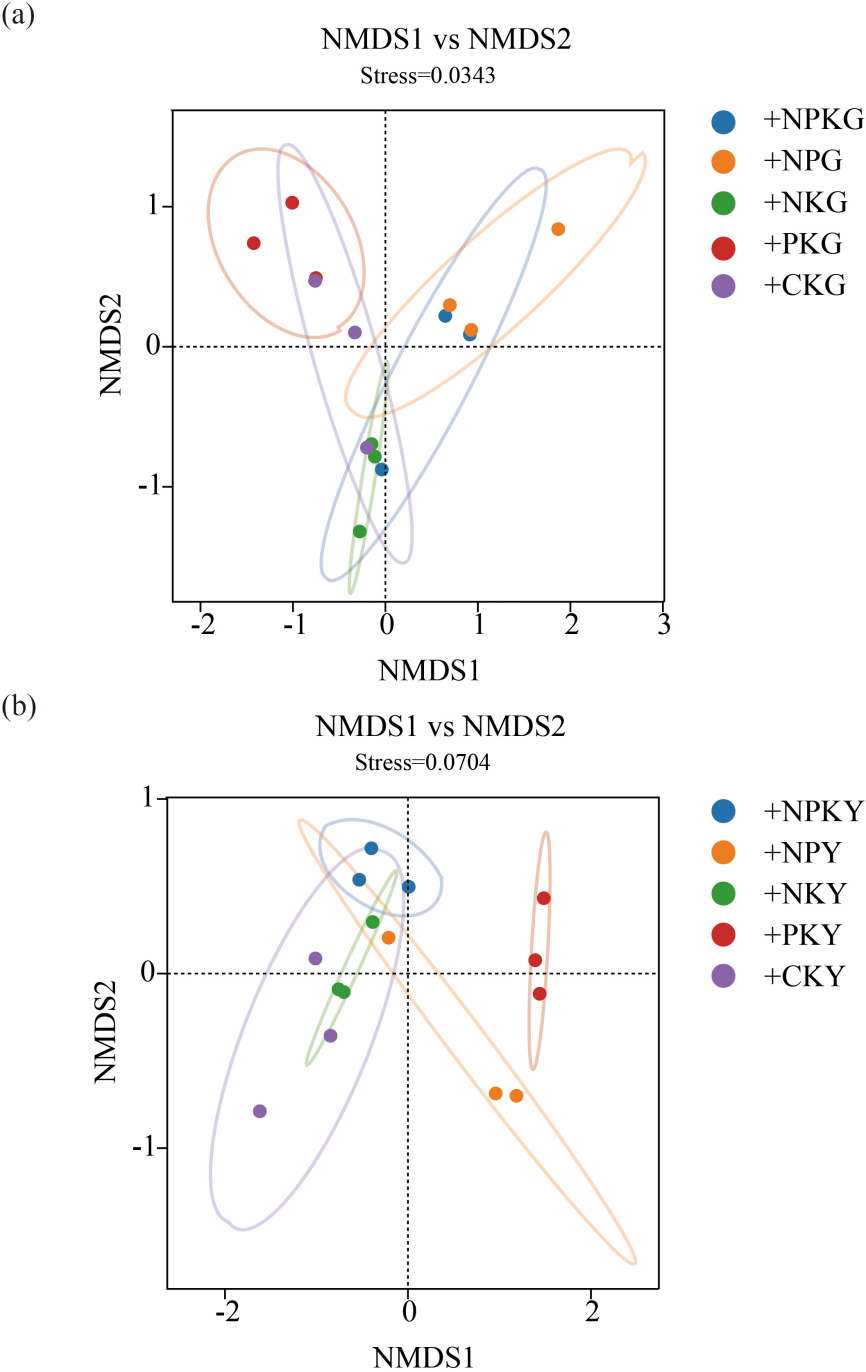
NMDS analysis of soil fungal communities in different fertilization treatments. **(a, b)** represent for alfalfa and awnless brome, respectively. In the treatment name, “G” represents *M. sativa* ‘Gannong’ No.3, while “Y” represents *Bromus inermis* ‘Yuanye’. CK (N 0 kg/ha, P 0 kg/ha, K 0 kg/ha), +PK (N 0 kg/ha, P 210 kg/ha, K 90 kg/ha), +N2K2 (N 120 kg/ha, P 0 kg/ha, K 90 kg/ha), +N2P2 (N 120 kg/ha, P 210 kg/ha, K 0 kg/ha), and +NPK (N 120 kg/ha, P 210 kg/ha, K 90 kg/ha).

**Figure 5:**
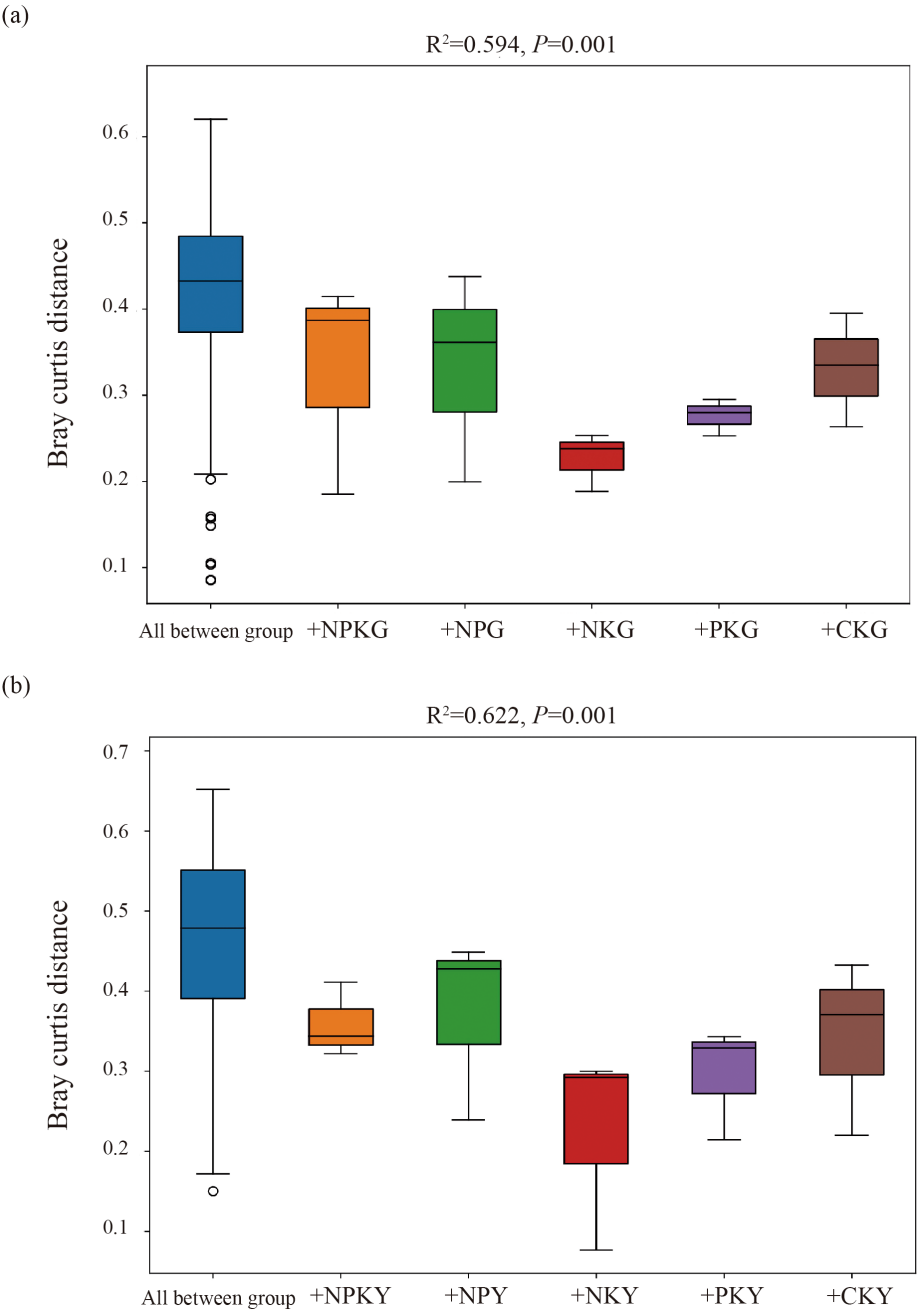
PERMANOVA test of soil fungal communities in different fertilization treatments. **(a, b)** represent for alfalfa and awnless brome, respectively. In the treatment name, “G” represents *M. sativa* ‘Gannong’ No.3, while “Y” represents *Bromus inermis* ‘Yuanye’. CK (N 0 kg/ha, P 0 kg/ha, K 0 kg/ha), +PK (N 0 kg/ha, P 210 kg/ha, K 90 kg/ha), +N2K2 (N 120 kg/ha, P 0 kg/ha, K 90 kg/ha), +N2P2 (N 120 kg/ha, P 210 kg/ha, K 0 kg/ha), and +NPK (N 120 kg/ha, P 210 kg/ha, K 90 kg/ha).

**Figure 6:**
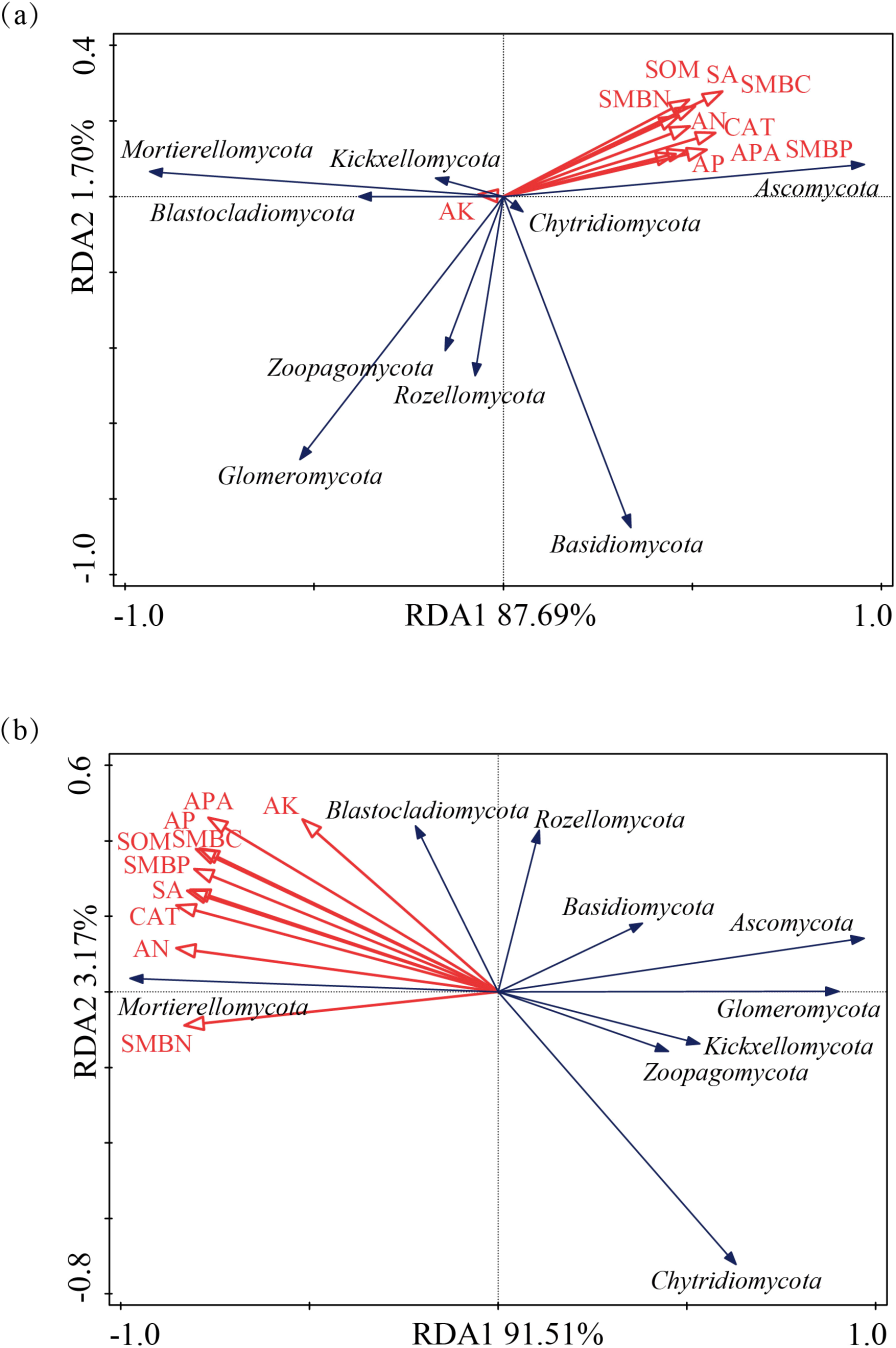
Redundancy analysis (RDA) of the relationships between microbial composition and soil environmental factors. **(a, b)** represent alfalfa and awnless brome, respectively.

For the fungal community, in alfalfa soil, +PK treatment was similar to the no fertilization treatment (**Figure 4**), but different from +NK, +NP, and +NPK treatments (PERMANOVA test, R²=0.594, *P*=0.001) (**Figure 5**). In the rhizosphere soil of awnless brome, there were differences between the +PK treatment and other treatments (**Figure 4**), while the clustering of fungal communities among other treatments was similar (PERMANOVA test, R²=0.622, *P*=0.001) (**Figure 5**). RDA analysis of soil with different fertilization treatments revealed that there was a significant effect of soil factors on fungi. As shown in **Figure 6a**, the explained amounts of the first and second axes were 87.69% and 1.7%, respectively. Soil SA (*P*=0.028), AK (*P*=0.01), and CAT (*P*=0.044) were the key environmental factors influencing the structure of the inter-root soil fungal community in alfalfa, which explained 29.6%, 30.9% and, 12.8% of the variance, respectively (**Table 3**). Among them, AK was highly significant and positively correlated with Mortierellomycota and Kickxellomycota; other factors were significantly positively correlated with Ascomycota and Chytridiomycota. As shown in **Figure 6b**, the explained amount of the first and second axes were 91.51% and 3.17%, respectively, and soil CAT (*P*=0.002) and SOM (*P*=0.038) were the key environmental factors influencing the structure of the inter-root soil fungal community in awnless brome, explaining 66.7% and 9% of the variance, respectively (**Table 4**). Also, there was a highly significant positive correlation between Mortierellomycota and Blastocladiomycota.

**Table 3:** Simple-term effects of environmental variables based on Monte Carlo permutation tests from the redundancy analysis (Alfalfa).

Name	Explains %	Contribution %	pseudo-F	*P*
SA	29.6	33.0	5.5	0.028
AK	30.9	34.3	9.4	0.010
CAT	12.8	14.2	5.2	0.044
SMBC	1.9	2.1	0.7	0.404
SOM	2.8	3.1	1.1	0.314
AN	1.4	1.5	0.5	0.468
APA	7.4	8.2	3.9	0.094
SMBP	1.9	2.1	1.0	0.368
AP	0.6	0.7	0.3	0.66
SMBN	0.7	0.8	0.3	0.684

**Table 4:** Simple-term effects of environmental variables based on Monte Carlo permutation tests from the redundancy analysis (Awnless brome).

Name	Explains %	Contribution %	pseudo-F	*P*
CAT	66.7	69.5	26.1	0.002
SOM	9.0	9.4	4.5	0.038
APA	1.6	1.6	0.8	0.434
SMBC	2.4	2.5	1.2	0.312
SMBN	3.7	3.8	2.0	0.172
AP	7.8	8.1	7.1	0.014
AN	1.7	1.8	1.7	0.230
SMBP	1.5	1.6	1.6	0.228
SA	0.7	0.7	0.7	0.504
AK	1.0	1.1	1.0	0.366

## Discussion

### Effects of fertilization on soil nutrient content in legume-grass mixtures

Rational fertilization can increase soil nutrients while promoting the sustained growth of agriculture (Sun et al., 2022). The influence of phosphorus, potassium, and nitrogen on legume-grass mixed grassland was an intricate combination of the elements instead of a single cumulative effect (Sardans and Peñuelas, 2015). This study obtained that, compared to no fertilization (CK), the contents of SOM, AN, AP, and AK in soil increased significantly under each fertilization treatment, with the balanced fertilization (+NPK) treatment being the most obvious, which was per previous investigations (Liu et al., 2023a). For each of the four fertilization treatments, the ranking of SOM and AP content was as follows: +NPK > +NP > +PK > +NK. About this result, previous studies have pointed out the application of phosphate fertilizer will increase AP content (Jin et al., 2021). The application of nitrogen, phosphorus, and potassium has been shown to significantly enhance the SOM content. This effect may be attributed to the synergistic influence of these nutrients on plant growth, which in turn stimulates soil microbial activity and leads to an increase in root exudates and plant litter (Guan et al., 2022). The SOM content was observed to be at its lowest under the +NK treatment. This phenomenon can be attributed to phosphorus deficiency, which adversely impacted the growth of alfalfa, subsequently diminishing its nitrogen fixation capacity and leading to a reduction in nitrogen levels within the soil (Xu et al., 2024). The AN content was found to be at its lowest under the +PK treatment, which may be due to alfalfa’s exclusive nitrogen fixation, which was not sufficient to meet the nitrogen requirements of legume-grass mixed grasslands. Moreover, Bromus inermis absorbed a significant amount of inorganic nitrogen from the soil, resulting in a decrease in nitrogen content (Bell et al., 2012). The soil AK content was ranked as follows: +NPK > +PK > +NK > +NP. The reason for this is that potassium fertilizer has a significant impact on the soil’s AK content (Jin et al., 2021).

### Effects of fertilization on soil enzyme activity in legume-grass mixtures

Soil enzymes are good catalysts for nutrient metabolism in the soil. Enzyme activity can reflect the extent of decomposition and transformation of substances in the soil and also reflect changes in soil fertility due to farmland management measures (Zhao et al., 2023). APA plays a significant role in the transformation of phosphorus in the soil, converting organic phosphorus into inorganic phosphate, which plants can absorb and utilize (Cui et al., 2024). The activity of soil sucrase facilitates the utilization of sucrose as an energy source by plants and soil bacteria through its hydrolysis into glucose and fructose (Yang et al., 2022). CAT serves to catalyze the decomposition of hydrogen peroxide, a byproduct generated during biological respiration, and the breakdown of organic matter, thereby mitigating its toxicity to soil organisms (Zhao et al., 2013). Compared to the CK treatment, the enzyme activity was significantly higher under the four fertilization treatments. This may be attributed to the fact that fertilization can stimulate the metabolic processes of plant roots, augment the production of root exudates, enhance microbial activity within the soil, and promote microbial proliferation, all of which contribute to an increase in soil enzyme activity (Chaudhari et al., 2020). The enzyme activity observed was significantly greater under the +NPK treatment compared to the three partial fertilization treatments. This phenomenon can be attributed to the inherently infertile soil located in the Hexi Corridor (Tan et al., 2022). The utilization of nitrogen-phosphorus-potassium fertilizer significantly enhanced the replenishment of vital soil mineral elements, thereby promoting the growth of alfalfa and awnless brome (Chen et al., 2022). The activities of SA, APA, and CAT were observed to be at their lowest levels under the +NK treatment. This phenomenon may be attributed to the dominant role of alfalfa within legume-grass mixtures. A deficiency in phosphorus significantly impairs the growth of alfalfa, thereby diminishing the nitrogen-fixing capacity of its nodules (Tahir et al., 2022). This reduction resulted in a deficiency of both nitrogen and phosphorus in the soil, which, in turn, led to a decrease in soil enzyme activity.

### Effects of fertilization on soil microbial biomass in legume-grass mixtures

Soil microbial biomass is viewed as a preliminary sign of changes in soil fertility and agroecosystem characteristics (Davies et al., 2022). SMBC is a sensitive component of soil organic carbon that can indicate soil microbial activity (Liu et al., 2023b). SMBP serves as a reservoir of bioavailable nutrients for plants and is crucial in the processes of phosphorus transformation and cycling (Hao et al., 2024). SMBN content indicates the influence exerted by soil microorganisms on nitrogen mineralization and fixation processes, in addition to representing the overall and inorganic nitrogen levels present in the soil (An et al., 2022). Fertilization strategies can have a positive effect on soil microbial biomass (An et al., 2023). The findings of this investigation revealed that the contents of SMBC and SMBP were ranked in the following order: +NPK > +NP > +PK > +NK > CK, Similarly, the content of SMBN was as follows: +NPK > +NP > +NK > +PK > CK. Notably, the levels of SMBC, SMBN, and SMBP were significantly higher under four fertilization treatments compared to the CK treatment, corroborating findings from previous studies. The application of phosphorus fertilizer improves its absorption of mineral nutrients, thereby increasing the content of SMBP in soil. Conversely, the treatment of phosphorus deficiency compels microorganisms to mobilize and release phosphorus, making it accessible for direct uptake and utilization by plants, which subsequently results in a reduction of SMBP level (Wang et al., 2024b). The utilization of nitrogen fertilizers has the potential to enhance the nitrogen levels in the soil, promote the decomposition of soil organic carbon by vegetation, supply adequate materials and energy for microbial activity, facilitate the mineralization of soil nutrients, and elevate the concentration of SMBN (Liu et al., 2024c). Furthermore, the trends in microbial carbon, nitrogen, and phosphorus content in leguminous and gramineous plants were consistent with the application of fertilizers. This consistency may be attributed to the differing root structures of legumes and gramineous plants, which facilitate the flow of nutrients from fertilizers between them, thereby promoting efficient resource utilization. This further underscores the advantages of legume-Grass mixtures in nutrient management (Cheng et al., 2023; Singh et al., 2023).

### Effects of fertilization on soil fungal community in legume-grass mixtures

The interaction between plants and microorganisms forms a complex ecological synergistic network, with these associated microorganisms playing a crucial role in the growth and development of plants (Hu et al., 2024). Depending on the type of fertilizer used in management practices, the relative abundance of dominant bacteria in the soil may change, although its overall composition remains stable (Sun et al., 2015). Our study found that the dominant phyla were Mortierellomycota, Ascomycota, Basidiomycota, and Chytridiomycota, among which Mortierellomycota and Ascomycota accounted for 82.2%-92.3%. Mortierellomycota has the ability to dissolve mineral phosphorus in the soil and enhance the content of soil nutrients by synthesizing and secreting oxalic acid (Zhou et al., 2018). As a result, Mortierellomycota exhibited increased activity in soils characterized by lower phosphorus concentrations, thereby contributing a greater quantity of bioavailable phosphorus to the soil ecosystem. The findings of this research indicated that the relative abundance of Mortierellomycota in the rhizosphere soil of alfalfa was greater in the absence of phosphorus treatment, corroborating the results of earlier investigations. Conversely, in the rhizosphere soil of gramineous forage grasses, a contrasting trend was observed, with rankings of +NP > +NPK > +NK > +PK > CK. This phenomenon was likely attributable to the application of nitrogen fertilizers, which significantly enhanced aboveground biomass. Such an increase created a conducive environment for the proliferation of Mortierellaceae, whose abundance was found to be greater in soils with high organic matter content (Jiawen et al., 2022). Ascomycota is recognized as the most prevalent and diverse group among eukaryotic organisms, playing a significant role in the decomposition of organic materials. Furthermore, it constitutes the primary fungal assemblage found in soils enriched with organic matter (Jiang et al., 2022). It typically exhibits rapid growth in conditions characterized by low soil fertility (Sun et al., 2023). This research indicated that the abundance of Ascomycota in the rhizosphere soil of gramineous forage was highest in the non-fertilization treatment, thereby corroborating earlier findings. In contrast, the abundance of Ascomycota in the rhizosphere soil of alfalfa exhibited a different pattern, likely due to the plant’s tendency to establish a symbiotic relationship with nitrogen-fixing bacteria (Clúa et al., 2018). The introduction of fertilizers may disrupt this symbiotic association and alter the composition of the microbial community (Klinerová and Dostál, 2020). Overall, the composition of the fungal community in legume-grass mixed soil subjected to various fertilization treatments exhibited similarities; however, there were significant differences in the relative abundance of individual fungal species.

### Analysis of environmental driving factors of soil fungal community

Fungi in the rhizosphere soil of Leguminosae and Grass become less abundant when nitrogen fertilizer was added; the +NPK treatment had the lowest fungal richness. When nitrogen fertilizer is absent, the breakdown of soil organic matter and plant root exudates are the primary sources of nitrogen in the soil (Feng et al., 2024). This nitrogen balance promotes fungal growth since excessive nitrogen concentrations can prevent some fungi from growing, which lowers community richness when nitrogen is added (Cadot et al., 2023). The right balance of nitrogen, phosphorus, and potassium can boost soil enzyme activity, support the growth of plant roots, and increase the capacity of microorganisms to break down organic matter. As a result, the +PK treatment shows the greatest diversity of fungal species, while the balanced fertilization treatment (+NPK) provides the best soil functionality (Wei et al., 2024). According to this study, the use of phosphorus fertilizer increased the variety of the alfalfa rhizosphere because it may encourage the growth and development of legume roots and raise their biomass and surface area. More attachment sites and nutrient supplies were made available by the expanded roots, which promotes fungal colonization and diversity (Bai et al., 2017).

Furthermore, we established that soil characteristics exert a considerable influence on fungal communities (Kruczyńska et al., 2023). RDA analysis of soil with different fertilization treatments revealed that soil SA (*P*=0.028), AK (*P*=0.01), and CAT (*P*=0.044) were the primary environmental factors influencing the structure of the inter-root soil fungal community in alfalfa (**Table 3**), consistent with the results of previous studies (Aslani borj et al., 2022). Among them, SA was intricately associated with the nutrient composition of soil and was utilized by soil organisms through the hydrolysis of sucrose, thereby supplying energy. This process serves as an indicator of the utilization of soluble substances within the soil, as well as the accumulation and transformation of soil nutrients (Wang et al., 2023). The activity of SA can alter the ratio of sugars present in the rhizosphere, which can either encourage or hinder the growth of certain fungal groups, ultimately influencing the makeup of fungal communities (Francioli et al., 2021). Furthermore, plants may regulate SA activity, which subsequently impacts the colonization of particular fungal groups (Ampt et al., 2022). The availability of potassium may significantly contribute to enhancing the symbiotic relationship between legumes and rhizobia (Liu et al., 2022). Moreover, the availability of potassium influences the types and amounts of root exudates produced by plants, which serve as vital carbon and energy sources for soil microbes. Various plants have distinct requirements and abilities to absorb potassium, leading to variations in their root exudates. This, in turn, impacts the composition of fungal communities in the rhizosphere (Xiao et al., 2022). Similarly, CAT was crucial for the symbiotic nitrogen fixation process involving legumes and rhizobia. These enzymes assist rhizobia in eliminating reactive oxygen species generated during nitrogen fixation, thereby safeguarding the functionality of nitrogenase. This protective role enhances the nitrogen fixation efficiency of rhizobia, ultimately supplying a greater amount of nitrogen to the legumes (Brewin, 1984). Variations in CAT activity influence the rate of nutrient release and their efficacy, contribute to signal transduction and the exchange of materials between plants and microorganisms, and facilitate the decomposition of organic matter. These variations also alter the competitive dynamics among different fungal species, ultimately impacting the structure of the community (Liu et al., 2024b; Jiang et al., 2024). Meanwhile, the positive correlation between AK and Mortierellomycota and Kickxellomycota suggests that these taxa may be susceptible to phosphate fertilizers (Liu et al., 2020). Soil CAT (*P*=0.002) and SOM (*P*=0.038) were identified as the key environmental factors affecting the structure of the inter-root soil fungal community in awnless brome (**Table 4**). Catalase converts hydrogen peroxide into oxygen and water, preserving the cellular redox balance (Gao et al., 2023). Reactive oxygen species generated during gramineous plant metabolism can be eliminated by catalase, which also produces an environment that is conducive to fungal development (Ihtisham et al., 2023). Grasses have the potential to locally elevate reactive oxygen species (ROS) as they supply carbon sources to fungi through the secretion of organic acids, sugars, and phenolic compounds from their roots (González-Pérez et al., 2018). In response to the accumulation of hydrogen peroxide, grasses enhance the activity of CAT, thereby alleviating stress within the microbial community and preserving the stability of the rhizosphere microenvironment (Zhu et al., 2025). The Grass were more reliant on the nitrogen source that soil organic matter provides than leguminous plants (Morizet-Davis et al., 2023). The primary influencing factor was soil organic matter since it is a significant source of nutrients for Grass rhizosphere fungi. Moreover, the observed factors exhibited a positive correlation with Mortierellomycota and Blastocladiomycota, both of which are categorized as saprophytic fungi (Bian et al., 2023; Chen et al., 2023). These fungi were capable of decomposing organic matter within soil ecosystems, thereby playing a vital role in the transformation and utilization of a variety of nutrients (Wu et al., 2022). This process enhances the availability of essential nutrients in the soil, subsequently fostering more favorable growth conditions for gramineous plants. Consequently, this indicates a positive correlation with several environmental factors.

Thus, there exist notable distinctions in the environmental factors influencing the Leguminosae and Grass families, attributable to three primary reasons: (i) Variations in physiological metabolism: Leguminosae possess the ability to fix nitrogen and engage in symbiotic relationships with rhizobium, resulting in differing nitrogen demands and utilization compared to Grass; (ii) Disparities in root structure and function: The root architecture of leguminous plants is more conducive to forming symbiotic associations with fungi, whereas the root structure of gramineous plants may be better adapted for growth in soils with elevated organic matter content; and (iii) Differences in nutrient acquisition and competition: Leguminous plants derive nitrogen through the process of nitrogen fixation, while gramineous plants predominantly depend on soil nitrogen. These distinctions ultimately culminate in varying sensitivities and requirements for different nutrient factors. The primary constraints of this experiment are temporal and spatial in nature, as it is limited to a single year of field research conducted in Wuwei.

## Conclusion

Compared to the CK treatment, the nutrient content, enzyme activity, and biological characteristics of rhizosphere soil were enhanced in other fertilization treatments. The balanced fertilization (+NPK) has the best soil function. The dominant fungal species in the rhizosphere soil of legume-Grass mixtures remained consistent, but fungal community structures varied under different treatments. RDA analysis identified soil SA, AK, and CAT as the main factors influencing the fungal community structure in alfalfa, while CAT and SOM were crucial for the fungal community in awnless brome. Consequently, the optimal fertilization strategy for establishing legume-grass mixed grassland in the sandy loam soil of the Hexi Corridor is determined to be N 120 kg/ha, P 210 kg/ha, K 90 kg/ha. Understanding fertilization needs of legume-Grass mixtures is key for agriculture, optimizing beneficial soil microorganisms, and improving pasture systems. However, more research is needed to clarify plant-microorganism-soil interactions.

## Data Availability

The datasets presented in this study can be found in online repositories. The names of the repository/repositories and accession number(s) can be found in the article/supplementary material.
